# Effects of dexmedetomidine dosage on the short-term cognitive function of elderly patients undergoing cardiac surgery

**DOI:** 10.1186/s12871-023-02315-6

**Published:** 2023-11-20

**Authors:** Jun Fang, Jia Yang, Mingyu Zhai, Qiong Zhang, Min Zhang, Yanhu Xie

**Affiliations:** 1 Department of Anesthesiology, The First Affiliated Hospital of USTC, Division of Life Sciences and Medicine, University of Science and Technology of China, Hefei, Anhui, 230001 China; 2 Department of Cardiovascular Surgery, The First Affiliated Hospital of USTC, Division of Life Sciences and Medicine, University of Science and Technology of China, Hefei, Anhui, 230001 China

**Keywords:** Dexmedetomidine, Infusion, Elderly patients, Cognitive function, Cardiac surgery

## Abstract

**Background:**

This study aimed to investigate the effects of perioperative dexmedetomidine (DEX) infusion rates on the postoperative short-term cognitive function.

**Methods:**

A total of 88 patients aged ≥ 60 years who underwent cardiac surgery from January 2022 to November 2022 at the First Affiliated Hospital of The University of Science and Technology of China (USTC) were included. Based on a single-center pilot analysis, patients were divided into two groups according to the rate of intraoperative DEX infusion, which started after tracheal intubation and continued until 1 h before extubation in the cardiac surgery intensive care unit. In Group L (*n* = 44), the infusion rate was 0.1–0.5 µg/kg/h (low-dose group), whereas in Group H (*n* = 44), the infusion rate was 0.5–0.9 µg/kg/h (high-dose group). Clinical outcomes were then compared between the groups. The Mini–Mental State Evaluation (abbreviated as MMSE_1_, MMSE_2_, MMSE_3_, and MMSE_4_) scale was used for the assessment of cognitive function, which was conducted on postoperative Days 2 (T_1_), 7 (T_2_), 14 (T_3_), and 28 (T_4_), with the score from postoperative Day 2 (MMSE_1_) considered as the primary observation.

**Results:**

Patients in Group L had higher MMSE_1_ scores compared to those in Group H (26.0 [24.0, 27.0] vs. 24.5 [22.0, 26.0], *p* = 0.046), and there was no significant difference in the scores between the groups at all subsequent time points. Group H exhibited a higher incidence of hypotension and bradycardia compared to Group L (*p* = 0.044 and *p* = 0.047, respectively).

**Conclusions:**

Compared to a high dose (0.5–0.9 µg/kg/h) of DEX infusion, a low-dose (0.1–0.5 µg/kg/h) infusion started after induction of anesthesia and continued until 1 h before extubation improved postoperative cognitive function scores on postoperative Day 2 in patients aged 60 years and older.

**Trial registration:**

URL: www.chictr.org.cn with registration number ChiCTR2100055093, registered on 31/12/2021.

## Background

Postoperative cognitive dysfunction (POCD) is a common complication that typically occurs within 1–3 months after surgery. Factors affecting postoperative cognitive function include patient age [1], cerebral perfusion pressure [2], cerebral microemboli [3], modes of cardiopulmonary bypass [4], systemic inflammatory response [5], preoperative depression [6], use of inhaled anesthesia [7], opioid administration [8], and sleep disturbances [9]. Dexmedetomidine (DEX) has been used in open heart surgery; however, its effect on postoperative cognitive function has yielded inconsistent results across different studies. These discrepancies can be attributed to differences in the objects being compared, cognitive assessment tools, and the infusion dose of DEX [10–12]. The optimal DEX infusion rate during cardiac surgery remains unknown. In a meta-analysis, Duan et al. [13] found that if the occurrence of delirium was the only primary endpoint, an optimal strategy might involve a loading dose ranging from 0 to 0.5 µg/kg, followed by a maintenance dose of 0.2 µg/kg/h. However, this conclusion is not universally applicable owing to the differences in research subjects, administration methods, and timing of drug delivery in clinical practice. Furthermore, it is difficult to inject DEX at a certain fixed dose. Therefore, it is more realistic to find the optimal range of DEX infusion rates to reduce POCD without side effects. According to the infusion rates of DEX listed in the previous anesthesia records from cardiac surgeries in our hospital, we found that the typical DEX infusion rate ranged between 0.1 and 0.9 µg/kg/h. After discussion with a statistician, we divided the DEX infusion rates into two groups with equally wide ranges: 0.1–0.5 µg/kg/h, classified as the low-dose group, and 0.5–0.9 µg/kg/h, classified as the high-dose group. The dosing strategy for DEX is tailored for each patient by the attending anesthesiologist based on their clinical experience, personal preference, and the patient’s condition. Given the significance of preventing cognitive impairment in elderly patients, a keen emphasis is placed on this aspect. The mini-mental state examination (MMSE) serves as a widely employed tool for evaluating cognitive function, and, in our study, the MMSE score on postoperative day 2 was designated as the primary study index.Our study did not focus on whether perioperative infusion of DEX reduces postoperative cognitive impairment. Instead, the main objective of this study was to compare the effects of different intraoperative DEX infusion doses on the cognitive function of elderly patients undergoing cardiopulmonary bypass (CPB) cardiac surgery. A secondary objective was to assess the impact of different perioperative DEX infusion doses on other complications and safety outcomes.

## Methods

### Study population and stratification

This trial was approved by the institutional review board of the First Hospital of the University of Science and Technology of China and registered at the Chinese Clinical Trial Registry with registration number ChiCTR2100055093(31/12/2021).

**Fig. 1:**
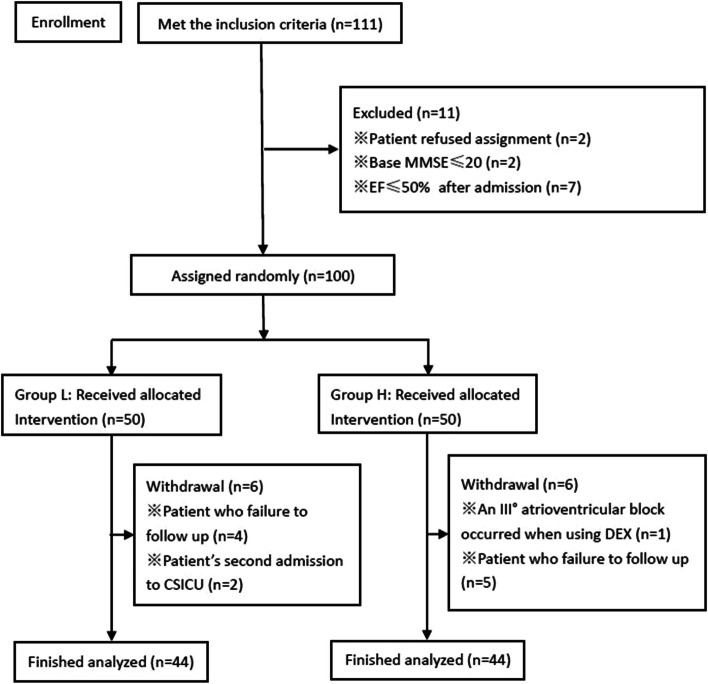
The technical flowchart of the clinical research on patients. MMSE, Mini-Mental State Evaluation; EF, ejection fraction; CSICU, cardiosurgery intensive care unit; DEX, dexmedetomidine

Out of 111 elderly patients who underwent cardiac surgery and received DEX infusion, 11 were excluded, 12 withdrew, and 88 were included in the study (Fig. 1). The patients were divided into two groups: Group L (*n* = 44), in which patients received a low-rate infusion of DEX at 0.1–0.5 µg/kg/h and Group H (*n* = 44), in which patients received a high-rate infusion of DEX at 0.5–0.9 µg/kg/h. The inclusion criteria were as follows: (1) having undergone cardiac surgery (valve or aortic root surgery) between January 2022 and November 2022; (2) age between 60 and 75 years; and (3) DEX infusion rate of 0.1–0.5 µg/kg/h or 0.5–0.9 µg/kg/h, continued until 1 h before postoperative extubation in the cardiac surgery intensive care unit (CSICU). The exclusion criteria were as follows: (1) preoperative disagreement or refusal to participate in the study; (2) pre-existing history of mental illness; (3) baseline MMSE (MMSE_0_) score ≤ 20; and (4) ejection fraction ≤ 50% after admission. The withdrawal criteria were as follows: (1) perioperative use of DEX that was not within the scope of the defined group parameters or the use of a loading dose; (2) a second admittance to CSICU; (3) incomplete postoperative follow-up data; (4) failure in patient data analysis; (5) Dex infusion for more than 36 h; and (6) III° atrioventricular block during Dex infusion.

All patients underwent cardiac surgery following standard anesthesia and CPB protocols and were routinely admitted to the CSICU for postoperative management. The types of surgery were categorized into valve surgery and aortic root surgery. Valve surgery included procedures such as isolated mitral, tricuspid, and aortic valve surgery; combined mitral and tricuspid valve surgery; and combined mitral and aortic valve surgery. Aortic root surgery included procedures such as Bentall surgery, Wheat surgery, and David surgery.

We collected and analyzed data related to age; sex; body mass index (BMI); MMSE_0_ (recorded on preoperative Day 1 [T_0_]); educational level (specifically, those with < 5 years of education); history of hypertension and diabetes; type and duration of surgery; preoperative ejection fraction (EF); hemoglobin level; benzodiazepine use; doses of sufentanil, propofol, and penehyclidine; and duration of DEX infusion (Table 1).

**Table 1:** Baseline clinical characteristics and intraoperative variables of Group L and Group H

Clinical variable	Group L, *n* = 44	Group H, *n* = 44	*P* value
Preoperative index			
Age (y)	66.0(62.3–71.8)	68.0(65.3–70.0)	0.313
BMI (kg/m^2^)	24.1(21.3–25.1)	24.2(23.0–28.0)	0.502
Gender (female)	18(40.9)	23(52.3)	0.285
MMSE_0_	28.0(26.0–29.0)	27.5(26.0–28.0)	0.259
ASA			0.560
III n (%)	6(13.6)	8(18.2)	
IV n (%)	38(86.4)	36(81.8)	
Type of surgery			> 0.999
Valve surgery n (%)	39(88.6)	39(88.6)	
Aortic root surgery n (%)	5(11.4)	5(11.4)	
EF (%)	64.2 ± 7.9	63.8 ± 6.9	0.797
Educational level < 5 years n(%)	20(45.5)	23(52.3)	0.522
CAS ≥ 50% n (%)	2(4.5)	10(22.7)	0.013
Smoking n (%)	0(0)	1(2.3)	> 0.999^#^
Hypertension n (%)	25(56.8)	13(29.5)	0.010
Diabetes n (%)	6(13.6)	6(13.6)	> 0.999
Haemoglobin(g/L)	126.4 ± 16.3	120.1 ± 16.7	0.076
Inoperative index			
CPB time (min)	119.5(100.3–155.0)	114.5(101.0-159.0)	0.897
Benzenediazepines use n (%)	38(86.4)	38(86.4)	> 0.999
Penehyclidine hydrochloride use n (%)	35(79.5)	39(88.6)	0.244
Length of surgery (min)	280.0(250.0-351.3)	290.0(255.0-347.5)	0.652
Anaesthesia time (min)	330.0(296.3-393.8)	345.0(320.0-400.0)	0.316
Sufentanil (ug)	350.0(311.8–395.0)	350.0(317.3-406.8)	0.698
Propofol (mg)	1100.0(989.5-1283.5)	1120.0(1000.0-1200.0)	0.776
DEX infusion time (h)	18.5(14.0–22.0)	20.0(14.0-26.8)	0.410

Data are shown as the mean ± standard deviation, number (percent), or median (interquartile range) as appropriate. ^#^Fisher's exact test

*BMI* Body mass index, *MMSE*_0_ baseline Mini-Mental State Evaluation, *ASA *American Statistical Association, *EF* Ejection fraction, *CAS* Carotid artery stenosis, *CPB *Cardiopulmonary bypass, *DEX *Dexmedetomidine

### Data collection

We recorded and collected relevant preoperative and intraoperative baseline data, as described above. The data also included various outcome indicators, such as MMSE score on postoperative Day 2 (MMSE_1_), Day 7 (MMSE_2_), Day 14 (MMSE_3_), and Day 28 (MMSE_4_); instances of intraoperative hypotension; duration of initial intubation; length of CSICU stay; incidence of postoperative delirium (POD); occurrence of myocardial infarction and stroke; length of hospital stay; and hospitalization-related mortality.

### Primary outcome measures

Our primary outcome was short-term cognitive function. We used the MMSE score recorded on postoperative Day 2 as the primary observation endpoint. Assessments were conducted in the CSICU or the cardiac surgery ward on this day. During inpatient evaluation, patients were assessed face-to-face by the researchers, whereas for out-of-hospital evaluations, the MMSE scale evaluations were conducted through WeChat video visits, combined with a patient self-help model. Uniform assessment forms were used for both in-hospital and out-of-hospital evaluations.

### Secondary outcome measures

The secondary outcomes for this study included the incidence of POD, intraoperative hypotension and bradycardia, length of hospital stay, and in-hospital mortality. The CSICU of our unit has a routine protocol for POD evaluation. To differentiate from the residual effects of anesthesia, delirium screening was started 24 h after cardiac surgery and performed twice a day. The Confusion Assessment Method Intensive Care Unit (CAM-ICU) scale was used to assess delirium every 12 h, specifically at 9:30 and 21:30. When a patient was discharged from the CSICU to the ward, the CAM was used to assess delirium every 12 h until postoperative Day 5. If delirium was not resolved by this day, the evaluation continued until discharge. The incidence of POD was defined as the percentage of patients who developed delirium during their hospitalization. Intraoperative hypotension was defined as systolic blood pressure below 80 mmHg lasting for at least 1 min or instances where the systolic blood pressure dipped below 80 mmHg at least twice. Intraoperative bradycardia was defined as a heart rate ≤ 60 beats/min. Myocardial infarction was defined as an increase in troponin I by more than 10 ng/mL during the postoperative CSICU and ward stays, along with higher than normal CK-MB, total CK exceeding 10%, and a new Q wave in the ECG lead in two or more consecutive measurements. Postoperative stroke was identified when its diagnosis was confirmed by a neurologist during postoperative CSICU and ward monitoring. CSICU duration was defined as the time of the patient’s stay in the CSICU, calculated in hours. Hospital stay was defined as the time from registration of patient admission to their discharge. In-hospital mortality was calculated as the ratio of deaths from CSICU admission to discharge relative to the total number of patients in the study group.

### Statistical analysis

#### Sample size calculation

This study aimed to compare the differences in the MMSE scores on postoperative Day 2 between the two study groups. Studies by Shaefi et al. [14] and Saczynski et al. [15] showed that the MMSE score on postoperative Day 2 is the lowest point, with the minimum clinically significant difference being 2 points after surgery. The pre-test results of 12 patients using low-dose infusion showed a mean and standard deviation of 24.4 ± 2.6 for the postoperative Day 2 MMSE scores. Assuming a difference test of the usage rate with a 5% two-tailed type I error rate (α = 0.05) and a test efficiency of 90%, the sample size was calculated using PASS 15.0 software, requiring 37 patients in each group. Considering a dropout rate of approximately 20%, a sample size of 46 patients per group was planned.

#### Statistical methods

Normally distributed continuous variables were described using mean ± standard deviation, non-normally distributed continuous variables were described as median (interquartile range), and categorical variables were described using percentages. The Mann–Whitney *U* test was used to compare the primary outcome indicators between the two groups; secondary outcome indicators, specifically the hours of initial intubation, CSICU stay, and duration of hospitalization; and baseline indicators such as age, BMI, MMSE_0_, CPB time, duration of surgery, anesthesia time, and infusion dose of sufentanil, propofol, and DEX infusion time. The Wilcoxon signed-rank test was used for within-group comparisons. Preoperative EF and hemoglobin were tested using an independent-sample *t-test*, and the remaining indicators were tested by Pearson chi-square test or Fisher’s exact test. Statistical analyses were performed using SPSS 22.0 (IBM, Armonk, NY, USA) or R software, and a two-tailed *p*-value < 0.05 was considered statistically significant.

## Results

### Baseline data and intraoperative parameters

The results of our patients are presented in Table 1. No statistically significant differences were observed between the two groups for the most analyzed indicators.

### Effects of different rates of DEX infusion on patient outcomes

**Fig. 2:**
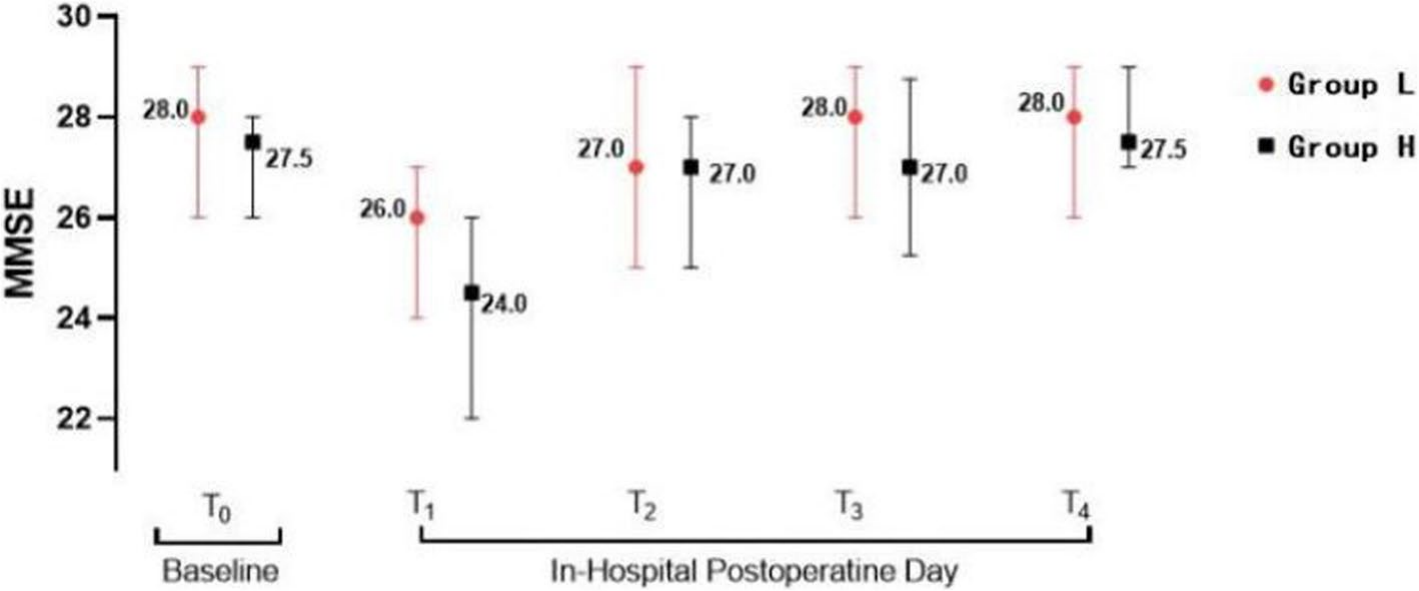
MMSE of patients at different time points during the study period. MMSE values are reported separately for the low-dose group (red dots) versus the high-dose group (black squares), and data are expressed as medians (interquartile spacing). MMSE, Mini-Mental State Examination; T_0_, on preoperative 1 day; T_1_, on postoperative 2 days; T_2_, on postoperative 7 days; T_3_, on postoperative 14 days; T_4_, on postoperative 28 days

Among the elderly patients included in this study, the MMSE_1_ score was lower in Group H compared to Group L after cardiac surgery (*p* = 0.046). No significant differences were observed between the MMSE_2_, MMSE_3_, and MMSE_4_ scores between the two groups. Group L showed lower incidences of both hypotension (*p* = 0.044) and bradycardia compared to Group H (*p* = 0.047). No statistically significant differences were observed in other secondary indicators between the two groups (Table 2). The MMSE score of both the groups decreased from the preoperative time point (T_0_) to postoperative Day 2 (T_1_; *p* < 0.001) and then increased on postoperative Day 3 (T_2_; *p* < 0.001). However, scores between T_2_, T_3_, and T_4_ remained consistent in their respective groups. These results can be seen in Tables 3 and 4, and Fig. 2.

**Table 2:** Postoperative outcomes in Group L and Group H

Clinical outcomes	Group L ,*n* = 44	Group H ,*n* = 44	*P* value
Primary outcome			
MMSE(T_1_)	26.0(24.0–27.0)	24.5(22.0–26.0)	0.046
MMSE(T_2_)	27.0(25.0–29.0)	27.0(25.0–28.0)	0.293
MMSE (T_3_)	28.0(26.0–29.0)	27.0(25.3–28.8)	0.344
MMSE (T_4_)	28.0(26.0–29.0)	27.5(27.0–29.0)	0.148
Secondary outcome			
POD n (%)	3(6.8)	3(6.8)	> 0.999^#^
Intraoperative hypotension n (%)	2(4.5)	8(18.2)	0.044
Intraoperative bradycardia n (%)	4(9.1)	11(25.0)	0.047
hours of initial intubation (h)	19.5(15.0–23.0)	21.0(15.0-27.8)	0.415
Myocardial infarction n (%)	0(0)	0(0)	
Stroke n (%)	0(0)	0(0)	
CSICU stay(h)	32.5(21.0–45.0)	40.5(21.3–61.3)	0.613
Hospital days (d)	24.5(20.-329.8)	23.0(18.0 -27.8)	0.207
Hospital mortality n (%)	0(0)	0(0)	

Data are shown as the mean ± standard deviation, number (percent), or median (interquartile range) as appropriate. ^#^Fisher's exact test

*MMSE* Mini-Mental State Evaluation, *T*_1_ on postoperative 2 days, *T*_2_ on postoperative 7 days, *T*_3_ on postoperative 14 days, *T*_4_ on postoperative 28 days, *POD* postoperative delirium, *CSICU* cardiosurgery intensive care unit

**Table 3:** Comparison MMSE of adjacent time points within the group L

Clinical outcomes		*P *value
MMSE(T_0_)	MMSE(T_1_)	
28.0(26.0–29.0)	26.0(24.0–27.0)*	< 0.001
MMSE(T_1_)	MMSE(T_2_)	
26.0(24.0–27.0)	27.0(25.0–29.0)**	< 0.001
MMSE(T_2_)	MMSE(T_3_)	
27.0(25.0–29.0)	28.0(26.0–29.0)	0.167
MMSE(T_3_)	MMSE(T_4_)	
28.0(26.0–29.0)	28.0(26.0–29.0)	0.064

Data are shown as the  median (interquartile range) as appropriate. *P <0.05,compared with the preoperative timepoint (T_0_) ,**P <0.05, compared with the postoperative 2 days (T_1_)

*MMSE *Mini-Mental State Evaluation, *T*_0_ the preoperative timepoint, *T*_1_ on postoperative 2 days, *T*_2_ on postoperative 7 days, *T*_3_ on postoperative 14 days, *T*_4_ on postoperative 28 days

**Table 4:** Comparison MMSE of adjacent time points within the group H

Clinical outcomes		*P* value
MMSE(T_0_)	MMSE(T_1_)	
27.5(26.0–28.0)	24.5(22.0–26.0)*	< 0.001
MMSE(T_1_)	MMSE(T_2_)	
24.5(22.0–26.0)	27.0(25.0–28.0)**	< 0.001
MMSE(T_2_)	MMSE(T_3_)	
27.0(25.0–28.0)	27.0(25.3–28.8)	0.092
MMSE(T_3_)	MMSE(T_4_)	
27.0(25.3–28.8)	27.5(27.0–29.0)	0.281

Data are shown as the  median (interquartile range) as appropriate. * *P* <0.05, compared with  the preoperative timepoint (T_0_) , **P <0.05, compared with the postoperative 2 days (T_1_).

*MMSE *Mini-Mental State Evaluation, *T*_0_ the preoperative timepoint, *T*_1_ on postoperative 2 days, *T*_2_ on postoperative 7 days, *T*_3_ on postoperative 14 days, *T*_4_ on postoperative 28 days

## Discussion

In our study, the high-dose group (0.5–0.9 µg/kg/h) showed a trend of relatively weaker neuroprotective effect compared to the low-dose group (0.1–0.5 µg/kg/h) on postoperative Day 2 (*p* = 0.046). However, animal studies by Zhou et al. [16] and Ma et al. [17] indicated that the neuroprotective effect of DEX is dose-dependent, with higher doses providing stronger neuroprotective effects. Zhang et al. [12] performed a prospective study on the effect of different doses of DEX on POCD in elderly patients, in which 80 patients undergoing laparoscopic colorectal cancer surgery were divided into four groups (*n* = 20), namely, control, D_1_, D_2_, and D_3_. While the control group received a loading dose of 0.5 µg/kg DEX, the D_1_, D_2_, and D_3_ groups received maintenance doses of 0.2 µg/kg/h, 0.5 µg/kg/h, and 0.8 µg/kg/h of DEX, respectively. The results showed that the high dose (0.8 µg/kg/h) afforded better neuroprotective effects compared to the low dose (0.20 µg/kg/h; *p* < 0.05). This difference may be attributed to the different durations of DEX intervention between the two studies. While Zhang et al.’s study included a short-term intervention of less than 3 h, our study had an overall median DEX infusion time of 19.0 (14.0, 24.8) hours for both groups. Another reason for the difference may be the increased incidence of hypotension in the high-dose group compared to the low-dose group in this study (*p* = 0.044). As documented in the literature, intraoperative hypotension may increase early cognitive dysfunction [18], diminishing the neuroprotective benefits in the high-dose group on postoperative Day 2. There was no difference in MMSE scores between the two groups at other time points (T_2_, T_3_, and T_4_). As shown in Fig. 2, the MMSE score at T_1_ was the lowest, but it returned to the baseline level at T_2_, T_3_, and T_4_. These data indicate that there was a transient decrease in MMSE scores on postoperative Day 2, which then returned to preoperative levels by postoperative Day 7 in both groups.

Moreover, this study showed that Group H had a slightly higher incidence of intraoperative hypotension (*p* = 0.044) and bradycardia (*p* = 0.047) compared to Group L. The anti-sympathetic effect (resulting in decreased blood pressure and slowed heart rate) was observed when DEX plasma concentrations were in the range of 0–0.7 ng/mL in Ebert et al.’s study [19], which showed that this anti-sympathetic effect could be amplified in a dose-dependent manner with the DEX infusion. Other secondary observations, including time to initial intubation, instances of myocardial infarction, stroke, duration of CSICU stay, and hospital mortality, did not significantly differ between the groups.

Our study has several limitations that should be considered. First, the *p*-value for the comparison of MMSE_1_ scores between the two groups on postoperative Day 2 was close to 0.5, rendering the evidence less convincing. Second, because this was a small single-center study, the limited sample size and the single study population limit the generalizability of the results. Third, the study only compared two different infusion dose groups without a blank control group; hence, our results cannot be simply attributed to a direct increase or decrease in postoperative cognitive function in our sample. Fourth, although the content of the in-hospital and out-of-hospital cognitive function assessments was similar, differences in the assessment methods may influence the results of the study.

## Conclusions

In patients aged 60 and older, a low-dose perioperative infusion of DEX (0.1–0.5 µg/kg/h) may offer better cognitive protection on postoperative Day 2 compared to a high-dose infusion (0.5–0.9 µg/kg/h). Additionally, to reduce the incidence of bradycardia and hypotension, a low-dose (0.1–0.5 µg/kg/h) DEX infusion during the perioperative period may be a better option.

## Data Availability

The data supporting this research can be obtained from the corresponding author upon reasonable request.

## Abbreviations

ASA: American Statistical Association
BMI: Body mass index
CAS: Carotid artery stenosis
CPB: Cardiopulmonary bypass
CSICU: Cardiac surgery intensive care unit
DEX: Dexmedetomidine
EF: Ejection fraction
MMSE: Mini–Mental State Evaluation
MMSE_0_: Baseline Mini–Mental State Evaluation
MMSE_1_: Postoperative Day 2 Mini–Mental State Evaluation
MMSE_2_: Postoperative Day 7 Mini–Mental State Evaluation
MMSE_3_: Postoperative Day 14 Mini–Mental State Evaluation
MMSE_4_: Postoperative Day 28 Mini–Mental State Evaluation
POD: Postoperative delirium
T_0_: The preoperative time point
T_1_: On postoperative Day 2
T_2_: On postoperative Day 7
T_3_: On postoperative Day 14
T_4_: On postoperative Day 28
