# Correction: Effects of dexmedetomidine dosage on the short-term cognitive function of elderly patients undergoing cardiac surgery

**DOI:** 10.1186/s12871-023-02399-0

**Published:** 2024-01-12

**Authors:** Jun Fang, Jia Yang, Mingyu Zhai, Qiong Zhang, Min Zhang, Yanhu Xie

**Affiliations:** 1 Department of Anesthesiology, The First Affiliated Hospital of USTC, Division of Life Sciences and Medicine, University of Science and Technology of China, Hefei, Anhui 230001 China; 2 Department of Cardiovascular Surgery, The First Affiliated Hospital of USTC, Division of Life Sciences and Medicine, University of Science and Technology of China, Hefei, Anhui 230001 China

**Correction: BMC Anesthesiol 23, 380 (2023)**

10.1186/s12871-023-02315-6

Following publication of the original article [1], the authors reported an error found in the affiliations, see below changes in bold.

**Incorrect**:

1. Department of Anesthesiology, Division of Life Sciences and Medicine, The First Affiliated Hospital of USTC, University of Science and Technology of China, Hefei, China.
2. Department of Cardiovascular Surgery, Division of Life Sciences and Medicine, The First Affiliated Hospital of USTC, University of Science and Technology of China, Hefei, China

**Corrected**:

1. Department of Anesthesiology, **The First Affiliated Hospital of USTC, Division of Life Sciences and Medicine**, University of Science and Technology of China, Hefei, Anhui 230001, China
2. Department of Cardiovascular Surgery, **The First Affiliated Hospital of USTC, Division of Life Sciences and Medicine**, University of Science and Technology of China, Hefei, Anhui 230001, China

The original article [1] has been updated.
